# Non-Traumatic Snapping Tendon on the Dorsal Aspect of the Thumb: A Diagnostic Challenge

**DOI:** 10.7759/cureus.14417

**Published:** 2021-04-11

**Authors:** Levin Kesu Belani, Shalimar Abdullah, Elaine Zi Fan Soh, Fauziana Abd Jabar, Zara Nasseri

**Affiliations:** 1 Orthopaedics and Traumatology, Fakulti Perubatan, Universiti Kebangsaan Malaysia, Kuala Lumpur, MYS; 2 Hand and Microsurgery, Fakulti Perubatan, Universiti Kebangsaan Malaysia, Kuala Lumpur, MYS; 3 Orthopaedics and Traumatology, Universiti Kebangsaan Malaysia Medical Centre, Kuala Lumpur, MYS; 4 Otolaryngology, Universiti Kebangsaan Malaysia Medical Centre, Kuala Lumpur, MYS

**Keywords:** tendon entrapment, trigger finger disorder, snapping finger

## Abstract

A snapping tendon on the dorsal aspect of the thumb is a rare condition as opposed to the common triggering on the volar aspect of the thumb. This condition is known as triggering of the extensor pollicis longus (EPL). A 21-year-old female presented with a clicking or snapping sensation that was felt on the dorsum of her thumb when it is extended. There was no history of trauma. She worked in an ice-cream parlor with repetitive scooping ice-cream motions. Her triggering immediately resolved on releasing the EPL fascia ulnar to Lister’s tubercle. Upon wake-up surgery, we could immediately confirm this. We recommend dynamic ultrasound as an investigation and do not recommend MRI. The surgical method of choice is either wake-up surgery or wide-awake local anesthesia no tourniquet (WALANT) surgery.

## Introduction

A snapping tendon on the dorsal side of the thumb has to be differentiated from the common trigger thumb on the volar aspect, which is due to thickening of the A1 pulley of the flexor tendon. This condition is named triggering of the extensor pollicis longus (EPL) and is rare.

The literature is limited on this condition. Only three published articles reported triggering of the EPL of the thumb in a non-traumatic situation attributed to ectopic bone formation [1], tendon nodule [2], and repetition [3]. Another two articles mentioned wrist pain mainly at the region of Lister’s tubercle [4,5].

Another four articles mentioned EPL triggering attributed to trauma [3,6-8]. We outline a case of dorsal triggering of the thumb with no history of direct trauma. This rare case puzzled us initially, and thus we would like to raise awareness of its diagnosis especially since there was no causative factor such as trauma.

## Case presentation

We report a case of a 21-year-old female who presented with a four-month history of clicking or snapping sensation felt on the dorsum of the right thumb when the thumb was extended. Initially, there was no pain, but two months later her pain score was 6/10 when she extended the thumb. She had no pain at rest. She had no history of definitive trauma and works in an ice-cream parlor. She was right hand dominant.

**Figure 1:**
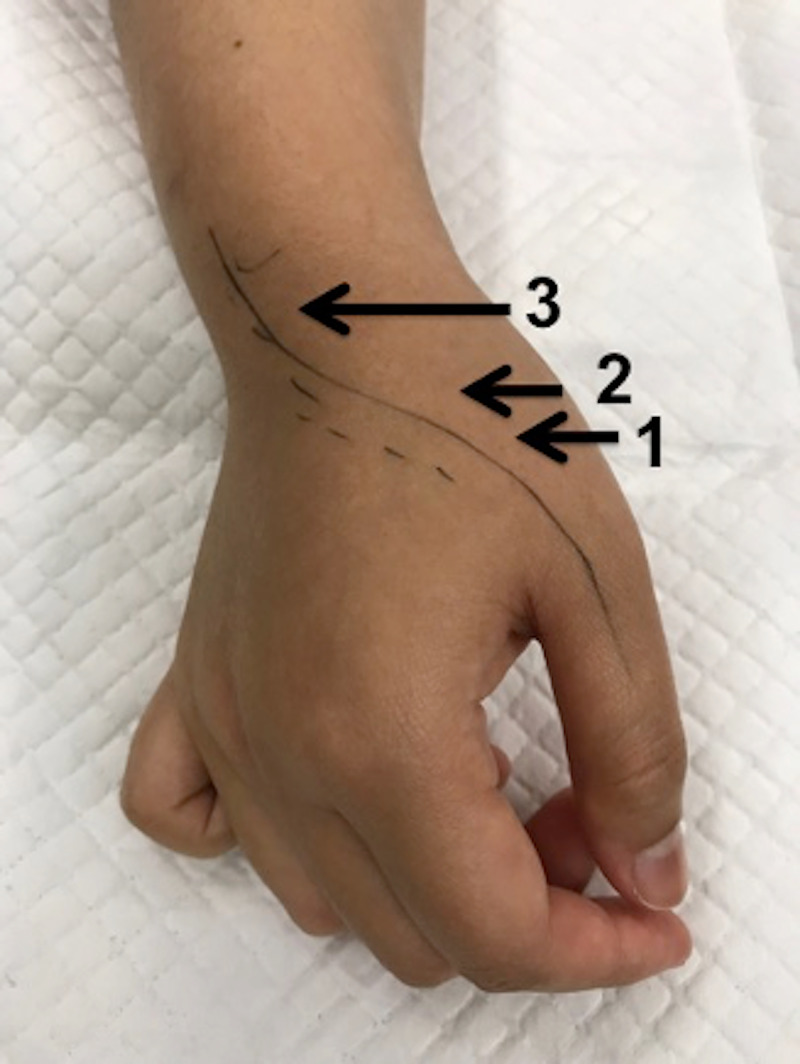
Triggering seen most obviously at the base of the first metacarpal bone (1) followed by the first carpometacarpal region (2) and least obvious at Lister’s tubercle (3).

During examination, triggering was seen in three different regions: at the base of the metacarpal, at the carpometacarpal joint (CMCJ) area, and at Lister’s tubercle (Figure 1). Out of these three areas, she mentioned that the triggering was more prominent at the CMCJ region than at Lister’s tubercle.

A dynamic ultrasound study reported displacement of the EPL tendon radially during flexion movements at the level of the first CMCJ. An MRI was also performed, but the findings were totally unrelated to our diagnosis. It reported first MCPJ subluxation with sprain of the radial collateral ligament. We decided to operate based on her symptoms and ultrasonographic findings.

Surgery was planned, and the patient offered either WALANT (wide-awake local anesthesia no tourniquet surgery) or wake-up surgery (anesthesia awareness surgery). This was to determine the exact area of pathology and to ensure her condition is fully resolved. The patient selected the latter.

**Figure 2:**
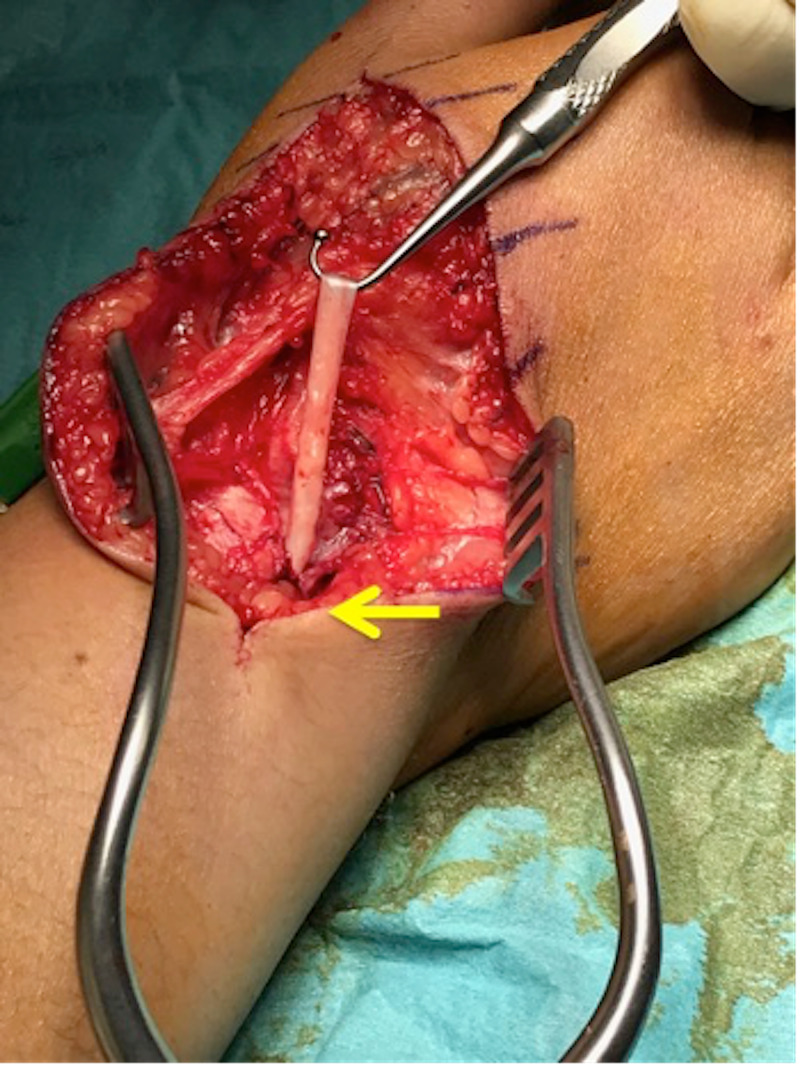
Top of the figure is the distal part of the hand. Bottom part of the figure is the forearm. The third compartment has been released and the extensor pollicis longus tendon is pulled out to show the tendon indentations.

**Figure 3:**
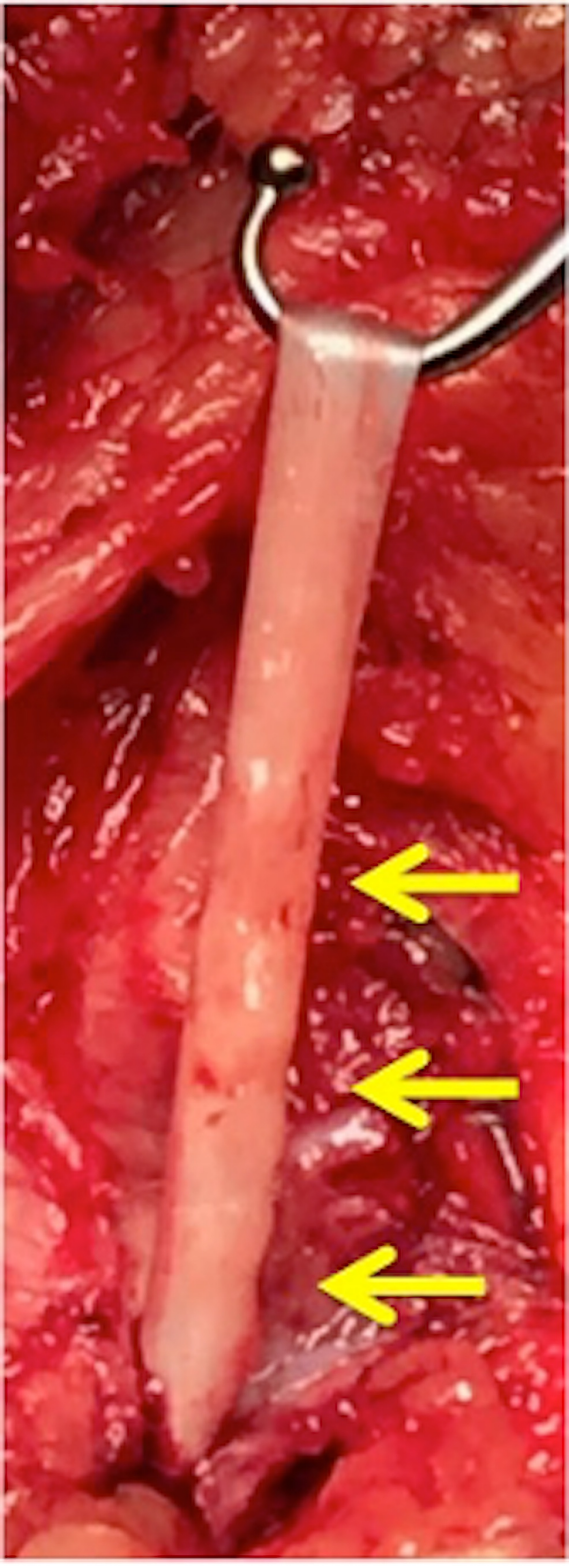
Closeup of the extensor pollicis longus showing three indentations (arrows).

Based on the history of the prominent triggering at the base of the first metacarpal region and the ultrasound results, we began the incision at the base of the first metacarpal region. However, no triggering was seen here. The incision was extended proximally to the CMCJ region, which again did not show any triggering. Finally, the incision was extended to Lister’s tubercle, and we were able to see triggering in this region (Figure 2). The patient was requested to repeatedly extend her thumb during surgery, which she complied with. Post-release of the fascia, her triggering resolved. The EPL tendon was seen to have three obvious areas of grooves or indentation in this area (Figure 3). This could have contributed to the three regions of triggering.

**Figure 4:**
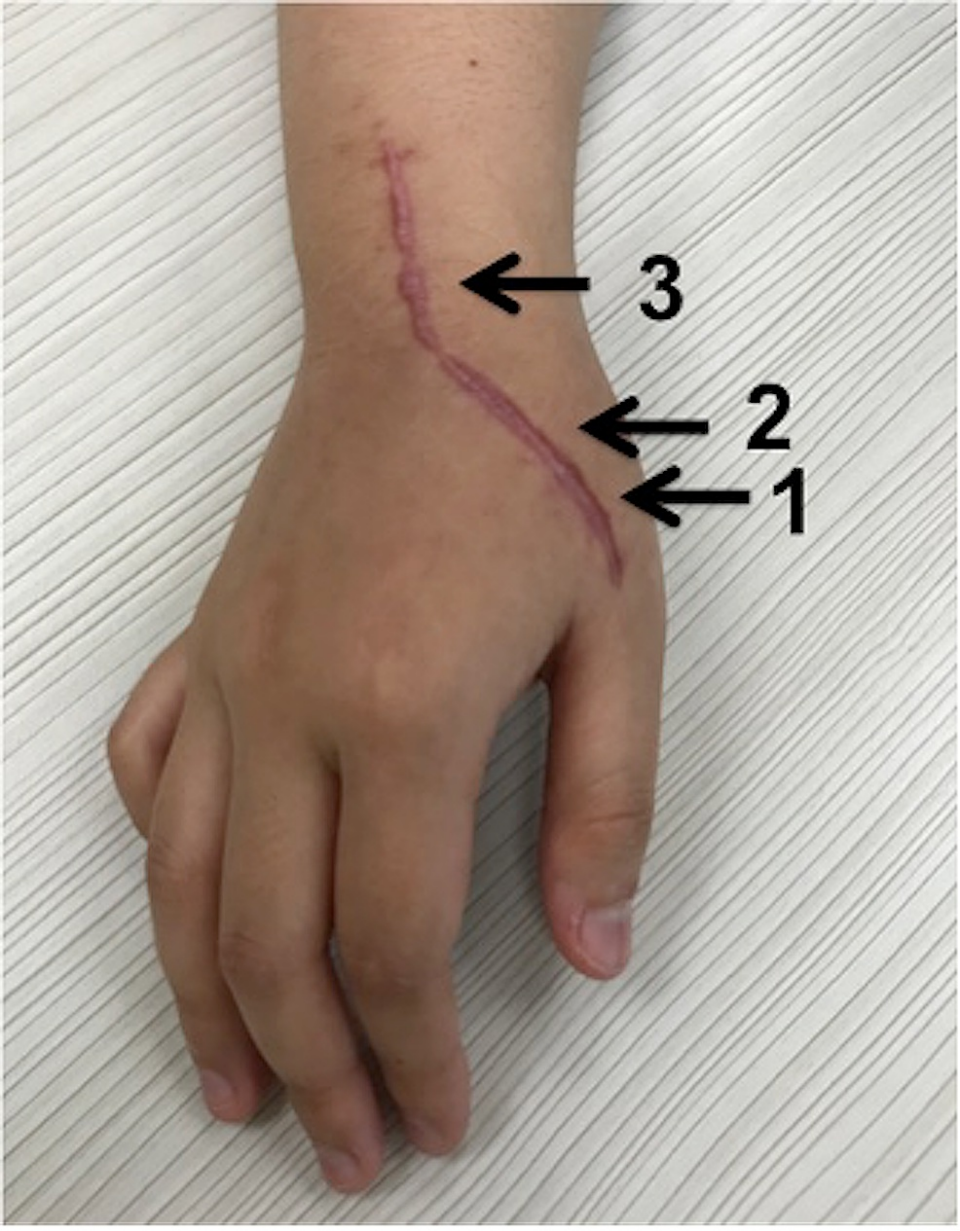
At three months post-surgery, a keloid scar was formed in the patient, and the marking 1, 2, and 3 were the positions where the triggering were seen most obviously intraoperatively.

At three months post-surgery, she developed a keloid scar (Figure 4) but she had no triggering or pain and was able to comfortably move her thumb.

## Discussion

To differentiate this condition from the more common trigger thumb, Kardashian et al. has proposed to use snapping for triggering on the dorsal side [3]. There are only eight published articles published in the literature on the snapping thumb. The articles are summarized according to traumatic or non-traumatic causes, age, gender, occupation, presence of triggering, and findings in Table 1. Kardashian et al. reported two cases, one was non-traumatic (repetitive) and the other traumatic [3].

**Table 1:** Comparison of the eight articles published on snapping or dorsal triggering of the thumb. *Kardashian et al. [3] reported two cases, one was traumatic and the other non-traumatic. EPL, extensor pollicis longus

Author	Year	Cases	Age, Gender, Occupation	Triggering	Findings
Non-traumatic causes					
Jackson et al. [1]	2007	1	34-year-old male (car factory worker)	Yes	Ectopic bone formation
Luenam et al. [2]	2010	1	42-year-old female (secretary)	Yes	Tendon nodule
Kardashian et al. [3]	2011	1*	26 year-old male (banker and drummer)	Yes	Slight EPL swelling
Abdullah et al. (this study)	2021	1	21-year-old female (ice-cream parlor worker)	Yes	Irregular EPL with three areas of indentation/nodules
Huang and Strauch [4]	2000	1	52-year-old male (uses weight lifting and rowing machine)	No	Swollen tendon, tight with serous yellow fluid
Mogensen and Mattsson [5]	1980	2	40-year-old female (factory worker); 26-year-old female (cashier)	No	EPL muscle extends into the distal third compartment
Traumatic causes					
McMahon and Posner [6]	1994	1	36 year-old female (physician)	Yes	Thickened extensor retinaculum, indentation in the EPL
Lanzetta et al. [7]	1995	1	25-year-old female (librarian)	Yes	Thickened area preventing tendon gliding freely
Ferreres et al. [8]	2008	3	25-year-old male (triggering seen); 32-year-old male (bricklayer) with tendon rupture; 42-year-old male with displaced tendon	Yes in one case	Direct injury to EPL at Lister’s tubercle with the wrist fully extended
Kardashian et al. [3]	2011	1*	44-year-old male	Yes	Discoloration and frayed tendon, loss of glistening surface

Kardashian et al. mentioned that repetitive motion could be a causative factor in their case report, which listed a banker who is also a drummer [3]. Other cases with possible repetitive motion are factory workers [1,5] and bricklayer [8]. Our patient was an ice-cream parlor worker and mentions repeated scooping motion of her right wrist and thumb. This could be classified as repetitive motion.

The area of impingement and hence triggering was noted to be between Lister’s tubercle and the base of the third metacarpal by Ferreres et al. [8]. When attempts at splinting and steroid injection failed, the surgical treatment was then to release the third compartment.

McMahon and Posner [6], Lanzetta et al. [7] and Kardashian et al. [3] additionally released the fourth compartment and incised the septum, separating them and allowing tendon gliding in a safe space followed by repair of this compartment to prevent bowstringing. Huang and Strauch incised the third compartment and only then re-sutured the floor of the third compartment to prevent resubluxation [4]. We incised the third compartment only and noted that the triggering was resolved. There was no subluxation seen and the tendon glided freely. We did not re-suture the floor nor released the fourth compartment.

Luenam et al. discovered an intratendinous nodule in the EPL. However, after releasing the EPL tendon, there was still triggering [2]. They then had to reroute the EPL superficially radial to Lister’s tubercle by suturing the extensor retinaculum.

Our problem was that the triggering was most obvious at the base of the metacarpophalangeal joint (MCPJ) followed by the CMCJ and least obvious at Lister’s tubercle. Hence, we initially started our incision at the base of the MCPJ and discovered that there was no triggering in this region. We then moved proximally to the CMCJ and finally to Lister’s tubercle before we noted the triggering. This resulted in a very long incision. We suggest hand surgeons to disregard the triggering at the CMCJ and MCPJ and focus at only Lister’s tubercle.

Our EPL tendon had three obvious grooves or indentations. Only McMahon and Posner described the indentation in the EPL [6]. Luenam et al. described an intratendinous nodule [2]. Perhaps our grooves and indentations were similar to three nodules. Kardashian et al. mentioned discoloration of the tendon with fraying [3] and Huang and Strauch mentioned a swollen tendon [4]. We encountered neither of those.

With regard to investigations, we do not advocate MRI but rather ultrasonography as the latter is a real-time investigation. We feel MRI is inaccurate and this is supported by De Maeseneer et al. who mentioned that due to the oblique orientation of the EPL this could result in a magic angle artifact [9].

## Conclusions

In conclusion, snapping of the thumb dorsally is a rare occurrence and the physician should be ever vigilant. Ultrasonography is the investigation of choice, with surgery to release the third extensor compartment ulnar to Lister’s tubercle.
